# Effects of a Weekend‐Long Field Course on Undergraduates' Confidence, Identity, and Belonging

**DOI:** 10.1002/ece3.72517

**Published:** 2025-12-04

**Authors:** Holly C. White, Katharine J. Ruskin, Debra M. Allen, Kayla B. McLagan, Sarah J. Nelson, Alison Jolley

**Affiliations:** ^1^ Ecology and Environmental Sciences, School of Biology and Ecology University of Maine Orono Maine USA; ^2^ Office of Institutional Research and Assessment University of Maine Orono Maine USA; ^3^ Appalachian Mountain Club Gorham New Hampshire USA; ^4^ Te Puna Ako ‐ Centre for Tertiary Teaching and Learning University of Waikato Hamilton New Zealand

**Keywords:** confidence, field course, research identity, sense of belonging

## Abstract

Field courses often serve as undergraduate students' first exposure to field‐based learning and are associated with a range of positive student outcomes including confidence in research skills, motivation to take more science classes, and learning gains. However, barriers such as family or work time commitments (i.e., most field courses are 7 weeks) can limit participation and inclusion, particularly for marginalized students. Much of the existing literature centers around traditional, multi‐week field experiences, creating a need to better understand outcomes related to shorter field courses. This study explored a weekend‐long field course designed to provide an early, low‐stakes research experience for undergraduate ecology and environmental science majors. Over 3 years and seven iterations of the course, we collected pre/post survey data (*n* = 104) in order to understand the efficacy of the short field course. After accounting for differences in student backgrounds, we found significant increases in research confidence, research identity, and sense of belonging. These findings suggested that even short field courses can have positive effects on students, similar to those resulting from multi‐week field experiences. This course can serve as a model for other universities and programs aiming to increase accessibility of field experiences and ecology as a career path.

## Graphical abstract

This study demonstrates that even a short, weekend‐long field course can significantly enhance undergraduate students' research identity, research confidence, and sense of belonging. By showing that short, low‐barrier experiences can produce meaningful outcomes, this work highlights an equitable model for expanding access to field experiences in ecology.

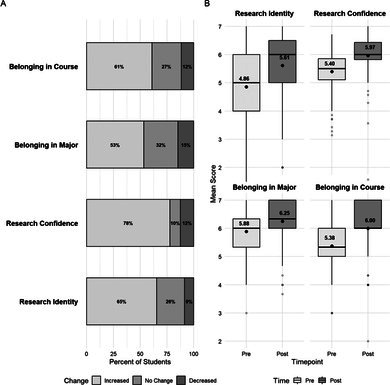

## 1 Introduction

Field courses serve as critical learning experiences that are unique from traditional classroom settings (Chitty and Hesp 2024; Elkins and Elkins 2007; Fedesco et al. 2020). Field experiences can promote cognitive learning gains (Easton and Gilburn 2012; Elkins and Elkins 2007), foster relationships among peers and faculty (Fedesco et al. 2020), motivate students to take more science courses (Shaulskiy et al. 2022), and foster a sense of place (Jolley et al. 2018). Many universities require field experiences as a part of their undergraduate curriculum in ecology, and practical field experience is valued by employers (Chitty and Hesp 2024). Additionally, the Ecological Society of America emphasizes the importance of fieldwork for strengthening ecology education in their Four Dimensional Ecology Education (4DEE) Framework (Klemow et al. 2019).

While field experiences are recognized as important, students still face barriers to participation and inclusion (Morales et al. 2020; Ruskin et al. 2024; Zavaleta et al. 2020). Students may face barriers related to accessibility (Hall et al. 2002; Stokes et al. 2019), lack of prior experience with nature and the outdoors (e.g., hiking, camping) (Fleischner et al. 2017), and costs associated with gear, course fees, and travel (Shinbrot et al. 2022; Smith 2004). Failure to reduce barriers to inclusion can lessen students' sense of belonging and reduce their interest in graduate studies (O'Brien et al. 2020), making this a critical issue of equity and inclusion in the discipline. Field courses are typically multiple weeks long (O'Connell et al. 2020) and some involve significant travel expenses (Giles et al. 2020), which can limit participation for students who cannot leave family or work obligations for extended periods of time (Fleischner et al. 2017; Giles et al. 2020; Ruskin et al. 2024; Shinbrot et al. 2022). Additionally, field course faculty face barriers including budget constraints and other logistical limitations (Fleischner et al. 2017) which are exacerbated in a multi‐week field course.

To address barriers related to traditional field courses, one option is to engage students with on‐campus field work (McCleery et al. 2005). However, research has shown that residential field courses have greater benefits than on‐campus courses (Shaulskiy et al. 2022), even if they incorporate on‐campus field work (Elkins and Elkins 2007). Much of the work on residential field courses has centered on positive outcomes in field experiences lasting one week or longer (Easton and Gilburn 2012; Nicotra et al. 2022; O'Connell et al. 2020; Shaulskiy et al. 2022), and the average length of a residential field course in ecology is seven weeks (O'Connell et al. 2020).

The limited research that exists on short residential field courses, or those < 1week in duration, where students are living at or near the field site where they are learning, suggests positive outcomes consistent with those of multi‐week residential field courses. One study found that a 10‐week on‐campus course involving two overnight trips as well as two additional day‐only trips (four total trips) promoted science identity (the extent to which they view themselves as scientists) among students when compared to students in a traditional lecture‐based course (Race et al. 2021). Race et al. (2021) attribute these gains to a “5 I's” design: inclusion (holistic support that lowers barriers, e.g., providing gear, skills), immersion (overnight field living/learning), interpersonal (intentional peer‐network and mentor relationships, emphasizing collaboration over competition), iteration (repeated trips and research projects that make growth visible), and inquiry‐driven (student‐generated questions and study designs). Similarly, another study found that multiple day field trips, plus two overnight trips, throughout the duration of a semester can support science identity (Treibergs et al. 2022) through unique opportunities to build relationships and a knowledge base that they could share with others. Other work comparing varying durations spent in the field found that more time, especially overnights, yielded stronger motivational and learning outcomes (Fedesco et al. 2020). These findings are promising in terms of reducing barriers of longer field courses such as costs, student work obligations, and logistics. However, there is a clear need to better understand student outcomes associated with short residential field courses (Shinbrot et al. 2022) as this literature remains sparse relative to research on traditional, multi‐week field courses.

To address this need, we previously put forth a detailed case of a standalone, weekend‐long, residential field course in ecology (Ruskin et al. 2024). This work outlined how the course model reduced barriers to participation in ecological field work and proposed the use of student survey instruments to measure relevant student outcomes.

Here, we aim to expand on this work by exploring student outcomes related to the course including research identity, research self‐efficacy (confidence), and sense of belonging, all of which play a role in student persistence and success. Belonging refers to the feeling of being a part of a social or academic community or group and is associated with retention, particularly for marginalized students (Bollen and Hoyle 1990; Tinto 1993; Walton and Cohen 2007). Identity and self‐efficacy, or confidence, are also key in fostering students' commitment to science disciplines and interest in science careers (Hunter et al. 2007; Robnett et al. 2015). Identity refers to the extent students view “science” or “research” as a core component of their self‐concept (Robnett et al. 2015), while self‐efficacy is the extent to which a student believes they can succeed in a certain domain (Bandura 1997). These constructs are often used to investigate undergraduate research outcomes and are the basis for the research questions we aim to assess. Specifically, we will address the following questions:

1. Can a short, weekend‐long field course affect students' research confidence, research identity, and belonging?
2. How do these outcomes differ across student demographics including income, first‐generation status, gender, and prior academic achievement?

## 2 Theoretical Framework

In this study, we conceptualize student success through sense of belonging, research confidence, and research identity, which are key drivers of student persistence (DeWitz et al. 2009; Tinto 1993). Sense of belonging and its importance in higher education have been extensively studied (Strayhorn 2019; Tinto 1993), and research shows it can occur at several levels from the small scale of belonging to a particular class to a broader sense of belonging within a campus or disciplinary community (Freeman et al. 2007; Hansen et al. 2024). In a traditional classroom setting, belonging can be fostered by relationships with peers and faculty (Zumbrunn et al. 2014), and similarly, at the campus level it is typically defined by students' perceived social support by faculty, peers, and staff (Strayhorn 2019). Field courses are well‐positioned to cultivate class‐level belonging (Shaulskiy et al. 2022), particularly because of their residential, collaborative structures (Race et al. 2021; Treibergs et al. 2022; Walsh et al. 2014).

Research self‐efficacy, or research confidence, is the extent to which a student believes they can be successful in research (Bandura 1997; Robnett et al. 2015). Prior work shows that participating in field courses produces greater gains in self‐efficacy, GPA, and higher graduation rates (Beltran et al. 2020). Bandura's theory of self‐efficacy identifies mastery experiences and vicarious experiences as two key influences (Bandura 1997), both of which are yielded through the hands‐on, immersive nature of field courses. Through iteration in field research projects and time spent in the field, students build confidence in their skillsets (Race et al. 2021). Lastly, research identity, the extent to which students view themselves as a researcher (Robnett et al. 2015), is influenced by one's performance of research practices (e.g., scientific communication, using tools), recognition (by oneself and others), and competence (knowledge and understanding of content) (Carlone and Johnson 2007). Recent work focused on field courses found that students' identities are impacted by their assessments of their competencies in comparison to their peers, perceived recognition, as well as shared experiences of overcoming challenges in fieldwork (Esparza et al. 2024). Altogether, the evidence suggests that field courses offer a unique context for students to develop belonging, confidence, and identity.

## 3 Methods

### 3.1 Field Research Experience in Ecology and Environmental Sciences (EES 217)

**FIGURE 1:**
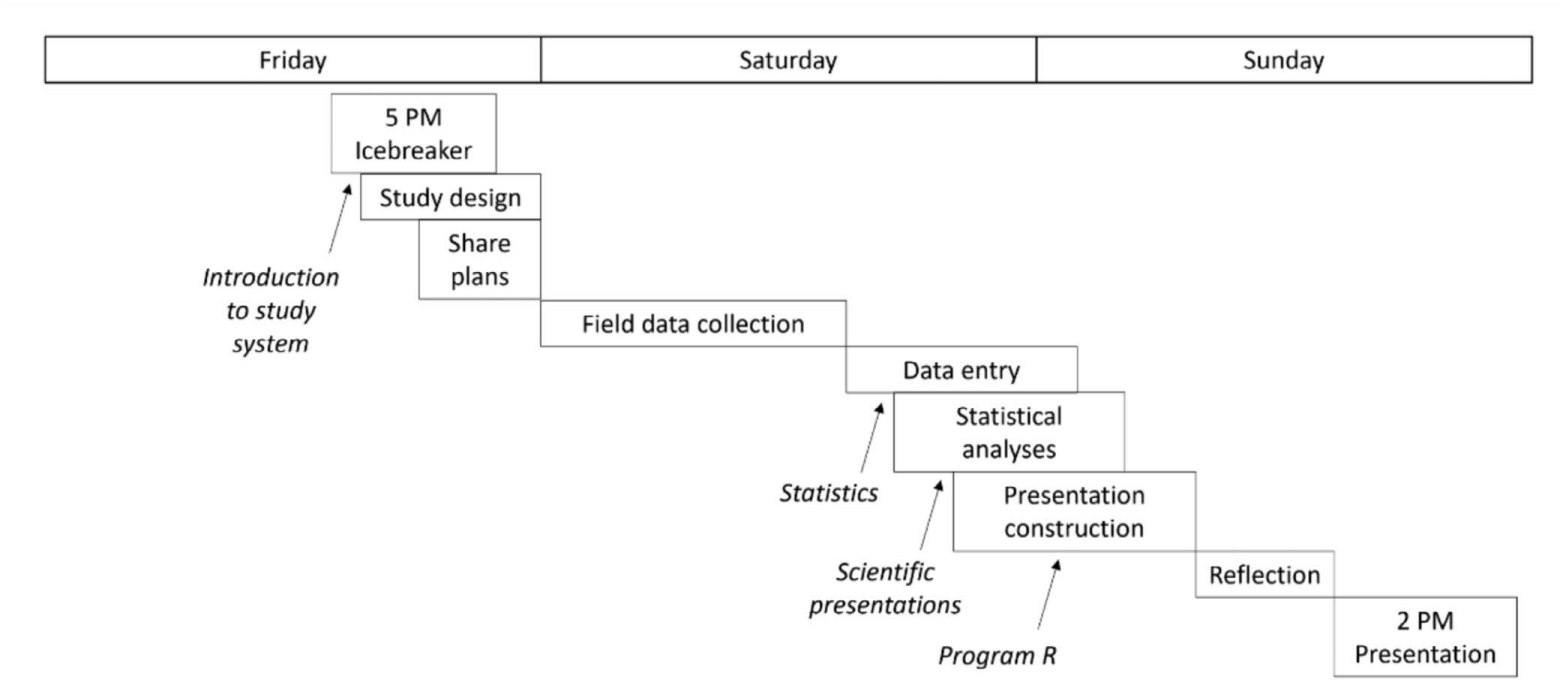
Course timeline. Reprinted from Ruskin et al. (2024). 2024 The Authors. Licensed under CC BY 4.0. https://doi.org/10.1002/ecs2.4912.

Field Research Experience in Ecology and Environmental Sciences (EES 217) is an undergraduate field research course in Acadia National Park that occurs over one weekend (3 days, 2 nights) (see Ruskin et al. 2024 for course syllabus). During this field experience, students developed research questions, collected and analyzed data, and presented their results in a 48‐h period (Figure 1). Though it was intensive, the course was one credit and pass fail, making it a low‐stakes experience. Additionally, it was a degree requirement for Ecology and Environmental Science (EES) majors, typically undertaken by sophomores and juniors. There was one pre‐trip meeting where the instructional team prepared the students with a packing list and class expectations including goals for the course, that students: (1) get experience in the field, (2) conduct an authentic research project based on a local environmental challenge, and (3) get to know their peers and instructors.

Course projects were co‐generated with the host research institution, the Schoodic Institute in Acadia National Park, based on interest‐holder needs and ongoing local research. After identifying broad project themes, students selected the one they were most interested in. Based on their interests the instructional team placed them into small groups (3–6 students per group). Within their chosen theme, students had the freedom to develop their own research questions. On the first night of arrival (Friday), each group developed a research question, formulated a hypothesis, and created a plan to investigate it the following day. Saturday was dedicated to field data collection in the morning and early afternoon, followed by data analysis and statistical methods workshops in the late afternoon and evening. By Sunday afternoon, students presented their findings to an audience that included interest‐holders, members of the public, as well as remote participants via Zoom. Examples of past research questions explored by students include: “How does distance from the tide line impact the number of shells deposited in different types of ecosystems?*”* and “What is the impact of human disturbances from trail walking on the plant diversity and moss coverage?*”*

Each offering of the course was taught by one lead instructor, one scientist from the host research institution, and at least two additional facilitators, who were either graduate or advanced undergraduate students. The role of each instructional team member was to facilitate the project for one student group. Facilitators served to support and encourage their assigned student group while allowing the students themselves to take the lead and shape the direction of their projects.

Ruskin et al. (2024) described how the course design of EES 217 reduces barriers to field work. The course was offered at little to no cost to students and essential gear such as mud boots was provided for students to borrow. Additionally, field sites were accessible by vehicle and did not require strenuous activity. Most importantly, it was only one weekend in duration, meaning that students did not have to take significant time away from other obligations such as work or caretaking roles. The present study builds on this work by assessing student data.

Course sections were split by student year (sophomore‐only section, junior‐only section) due to funding available for sophomore‐only research‐based courses. Throughout the study period, there were seven course sections offered which were led by three separate lead instructors. The course design (Figure 1) remained consistent across all sections as all instructors were provided the same instructional materials and guidelines.

Three out of the five authors on this manuscript served on the instructional team during the course sections surveyed for this study. Ruskin served as the lead instructor for five out of seven of the sections included in this study. McLagan and White served as learning assistants at separate times throughout the course's lifespan (McLagan, *n* = 5; White *n* = 2). The instructional team was careful to avoid undue influence and informed students that participation in the research study was voluntary, kept confidential from the instructor, and would have no impact on their standing in the course. For those who chose to participate, responses were anonymized using a randomly generated ID key by a researcher with no connection to the course or students. Allen, White, and Ruskin are involved in UMaine‐wide assessment of undergraduate research experiences, but the lead instructor (Ruskin) did not have access to any individual responses, even after deidentification. Finally, some authors had experience with the course prior to the study period. McLagan also participated in the course as a student before the research study began. Nelson developed and instructed the pilot of the course in 2015, and Ruskin continued development and instructed the course 2–3 times per year since 2016. This study was motivated by the authors' prior experiences with field‐based experiences and a shared interest in improving undergraduate ecology education. This work was approved by the IRB at the University of Maine (#2021‐07‐01).

### 3.2 Survey Instruments

The first part of the survey measured research confidence and identity (adapted from Robnett et al. 2015). This survey measure was originally developed for use within a university student population. The original survey consisted of three factors: research experience, science self‐efficacy (confidence), and science identity. For the present study, we used the science self‐efficacy and identity factors, which we relabeled research confidence and research identity to reflect minor wording changes we made. For example, for an item on the research self‐efficacy scale, we changed “Use scientific literature to guide research” to “Use research conducted by others to guide my research” to increase clarity and accessibility for novice students. The other items in this scale were not altered. Additionally, we made minor changes to the identity scale. Specifically, we reduced it from five items to two items to shorten the survey duration. Wording changes were minimal; for example, “I feel like I belong in science” was changed to “I feel like I belong in research.” These changes were made to better align with the course goals which were centered on the research process. Overall, there were nine total items: seven research confidence items and two research identity items. Below we report on measurement statistics.

Additionally, the survey measured sense of belonging to their major (EES) and their course (EES 217) (adapted from Bollen and Hoyle 1990). This instrument was originally developed and used on college students and has continued to be used across several diverse undergraduate institutions (Hausmann et al. 2007; Hurtado and Carter 1997; Hurtado et al. 2007). Each of the two survey scales (major and course) consisted of three items, for a total of six items. Students ranked each item on a 7‐point Likert scale with one being strongly disagree and seven being strongly agree. This part of the survey included questions such as “I feel a sense of belonging to my major/academic discipline” and “I see myself as part of my course community” (see Appendix S1 for the full survey).

### 3.3 Data Collection

To understand how student outcomes changed after completion of the field course, we administered pre/post surveys. These were collected from students over 3 years in seven iterations of the course (2021–2023). The pre‐surveys were distributed online in the week prior to the course, and post‐surveys were given at the end of the final day (Sunday) or the following day. On average, students completed the post‐survey 14 days after the course concluded.

Demographic information including first‐generation status, Pell Grant eligibility (students whose financial need deems them eligible for the federal Pell Grant program), gender, Honors program status, and cumulative GPA at the end of the term was provided to us by the university. Students were admitted to the Honors program based on their high school GPA and course rigor. Consequently, participation in the Honors College served as a proxy for academic preparedness for college. Data on race/ethnicity were provided but were not utilized due to the survey participants being 90% white. Our survey also asked students to report their gender in 2022 and 2023. However, in 2021 we did not include this question so we retrieved information on sex from the university demographic data which was reported as male/female. The gender question on our survey provided a broader array of options and allowed students to write in their gender if they did not identify with a provided option.

Recognizing the limitations of conflating sex with gender (Garvey et al. 2019), we merged the self‐reported gender data and the university sex data into a single variable for the purpose of comparison analyses against men. Additionally, we merged gender diverse students (*n* = 1) into this category as well, labeling the final category “Women and gender diverse”. Prior research shows that both women (Simon et al. 2017) and gender‐nonconforming students (Casper et al. 2022) are marginalized in STEM disciplines, which informed our decision to combine them into a single category.

### 3.4 Survey Measurement and Reliability

We used confirmatory factor analysis (CFA) with the lavaan package (Rosseel 2012) in RStudio (R Core Team 2021) to test whether items reflected their intended constructs and to assess reliability. A two‐factor CFA was used for research identity and confidence. We constrained the two loadings to be equal (tau‐equivalent) for the identity scale since it was two items to ensure it was identifiable and estimates were stable (e.g., Neff et al. 2021; Little et al. 1999). The research confidence loadings were estimated freely. To test fit, we used the comparative fit index (CFI), root mean square error of approximation (RMSEA), standardized root mean square residual (SRMR), and chi‐square (χ^2^). For reliability, we report the composite reliability (CR; Raykov 1997) which allows for unequal factor loadings (Rönkkö and Cho 2022). We also assessed convergent validity with average variance extracted (AVE) which shows the variance captured by the factor vs. error, and discriminant validity (to ensure identity and confidence were not redundant) with the latent correlation (φ) between constructs (Rönkkö and Cho 2022). With these, we checked that AVE was greater than the squared latent correlation (Fornell and Larcker 1981). Finally, we report on inter‐item correlation and Spearman‐Brown reliability for research identity as this is recommended for two‐item factors (Eisinga et al. 2013). Each belonging construct (course and major) was modeled as a single‐factor CFA. Since these were each three‐item surveys, they were just‐identified (df = 0), so fit indices were uninformative. To assess the quality, we used factor loadings and composite reliability (CR), as well as AVE for convergent validity.

### 3.5 Survey Analyses

Data analyses were conducted with R Statistical Software (R Core Team 2021) and RStudio (R Core Team 2021). To analyze each subscale, we calculated the mean of the corresponding survey items and treated this average as a continuous variable, consistent with the original use of these instruments (Bollen and Hoyle 1990; Robnett et al. 2015) and with survey research literature (e.g., Boone and Boone 2012). For example, belonging to major consisted of three survey items, so for each student we calculated the mean of the three items for the purpose of analyses.

We employed linear mixed effects models to examine whether there were significant pre/post changes in survey outcomes. The nlme package in RStudio was used (Pinheiro et al. 2022). Linear mixed effects models were chosen because our data are repeated measures (pre and post) and students are clustered by class section (non‐independence). Compared to other repeated measures techniques such as ANOVA, mixed effects models have advantages as they account for variance between class sections for the non‐independence of students in classes (Gueorguieva and Krystal 2004; Muhammad 2023). Additionally, they allowed us to control for demographic variables and test for interactions of these demographics (Jost and Jansen 2022).

All models included timepoint (pre vs. post) as a fixed effect and controlled for key demographic covariates, including gender, first‐generation status, Pell Grant eligibility (low income), honors status, and cumulative GPA at the end of term (log‐transformed and centered). To account for the hierarchical structure of the data and for the different instructors, we included a random intercept for class section, allowing for variation in survey outcomes across different sections. Additionally, nested student identifiers were used to model repeated measures at the individual student level.

Our first models (four; one per survey outcome) included all of the variables described as additive effects. In addition, to explore whether pre/post changes *differed* across demographics, we ran a second round of models with all interactions between time and demographic variables (global interaction model). Third, because of our small sample sizes in demographic categories, we also opted to run individual models where we added the five interaction terms into the models one at a time (20 models; see Appendix S3: Tables C1–C5).

## 4 Results

### 4.1 Sample and Response Rates

Across the 3 years of the study, 147 students completed the course and were given the opportunity to participate in the survey, of which 137 completed the pre‐survey and 112 completed the post‐survey. If students had duplicate surveys (e.g., took the post‐survey twice), we kept the first response and removed the later response. Student surveys were only considered complete if they participated in both the pre and post surveys (*n* = 108), as the two time points were critical for our statistical analyses. Additionally, due to our use of demographic data in the model, students needed to have complete demographic information, reducing the sample size to 104, or a 70% response rate (Table 1). Belonging to course and major were only measured in 2022 and 2023, reducing the sample size for models exploring those outcomes (see Table 3; *n* = 77).

**TABLE 1:** Number of students enrolled in the subject EES 217 course and surveyed by demographic.

Year	Enrolled	Surveyed (% response)	First generation	Pell eligible	Honors college	Women & gender diverse	Avg. GPA (End of term)
			(Surveyed/Enrolled)	(Surveyed/Enrolled)	(Surveyed/Enrolled)	(Surveyed/Enrolled)	(Surveyed/Enrolled)
2021	40	27 (67%)	6/9	2/6	4/6	15/19	3.48/3.35
2022	62	54 (87%)	5/7	4/5	8/9	37/43	3.49/3.45
2023	45	23 (51%)	6/9	5/10	3/10	17/34	3.49/3.48
Total	147	104 (70%)	17/25	11/21	15/25	69/96	3.49/3.42

*Note:* Values in each demographic column represent the number of students enrolled in that category and the number who completed the survey (Enrolled/Surveyed). GPA values reflect average GPAs for enrolled and surveyed students, respectively.

Across the 3‐year study period, EES majors were predominantly female comprising 61% of enrolled students. This trend was also reflected among course enrollees and survey respondents (Table 1). First‐generation students made up 18% of the major population and 16% of the survey respondents, indicating that they were well represented in the survey sample. While 23% of students in the major were in the Honors College, they accounted for 17% of course enrollees and 14% of survey respondents. Additionally, Pell grant‐eligible students (students who display significant financial need as determined by FAFSA, the Free Application for Federal Student Aid) were underrepresented in the survey as they made up 18% of the major population, 14% of EES 217 enrollees, but only 11% of survey respondents. Overall, the survey sample was representative of the course enrollees and the major, with slight underrepresentation of low‐income students. Proportions of students in each demographic group were relatively similar in each of the study years. Data on race/ethnicity were provided but were not utilized due to the survey participants being 90% white.

### 4.2 Survey Measurement and Reliability Results

A two‐factor CFA on the research confidence and identity pre‐survey data showed acceptable fit, *χ* ^2^(27) = 46.62, CFI = 0.968, RMSEA = 0.073, SRMR = 0.046, indicating the model represents the covariance structure reasonably well. Research identity had strong standardized loadings (0.88, 0.84), composite reliability (CR = 0.85), and average variance extracted (AVE = 0.74). This means that both items align with the construct and, on average, over 70% of their variance is shared with the latent factor. Research confidence showed moderate to high loadings (0.72, 0.67, 0.63, 0.66, 0.82, 0.81, 0.78), composite reliability (CR = 0.89), and AVE (0.53), indicating acceptable internal consistency and that the factor explains just over 50% of the item variance. Discriminant validity between identity and confidence was supported. The latent correlation (φ) was 0.60 (φ^2^ = 0.36), and each factor's AVE (0.74; 0.53) exceeded φ^2^, indicating that the two constructs are related but not redundant. Since identity is a two‐item scale, we also report on inter‐item correlation (*r* = 0.75) and Spearman‐Brown reliability (*ρ* = 0.85), which both indicate acceptable reliability. For sense of belonging, single‐factor CFAs indicated strong convergence and internal consistency: belonging to the course had high loadings of 0.85, 0.97, 0.95, CR = 0.95, AVE = 0.85, and belonging to the major had loadings of 0.73, 0.91, 0.89, CR = 0.88, AVE = 0.72. Most of the item variance reflects the intended belonging constructs.

### 4.3 Research Q1: Can a Short Weekend‐Long Field Course Affect Students' Research Confidence, Research Identity, and Belonging?

**FIGURE 2:**
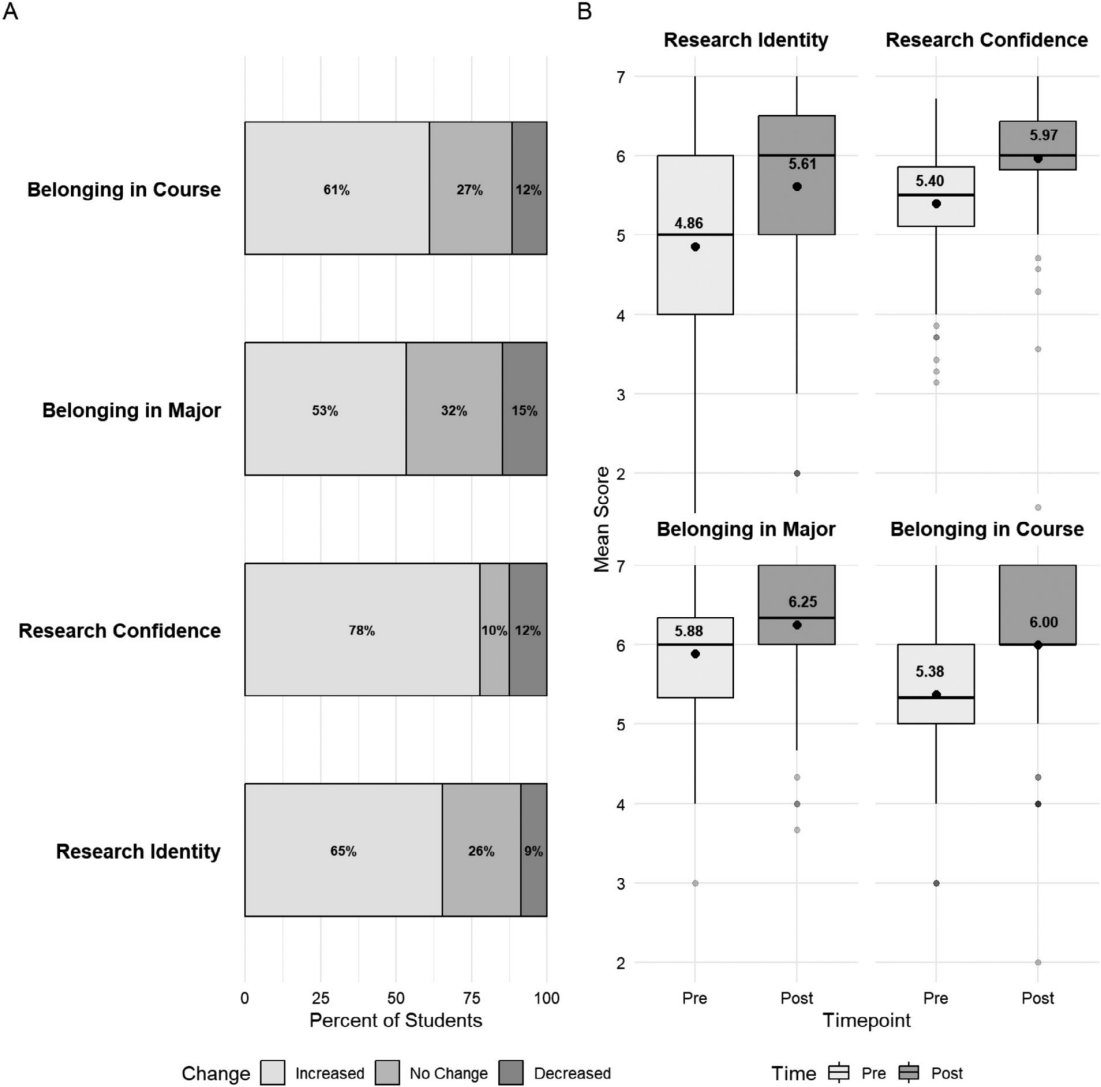
(A) Percentage of students who increased, decreased, and had no change from pre to post semester on each survey outcome, (B) Pre‐post means for each survey outcome.

Before running statistical analyses, we examined descriptive statistics including the percent of students who increased, decreased, and stayed the same from pre/post (Figure 2A), as well as pre/post means (Figure 2B). Descriptive statistics indicated positive shifts from the beginning to the end of the course. Specifically, 65% of students increased from pre to post on the research identity scale, 78% increased on research confidence, 53% increased on belonging to major, and 61% increased on belonging to course.

Linear mixed effects models showed that the fixed effect of time was significant across all four models that included variables as additive effects (Tables 2 and 3): research confidence (*B* = 0.57), research identity (*B* = 0.75), belonging to course (*B* = 0.62), and belonging to major (*B* = 0.37). Model fit statistics suggest that the random effects accounted for a large amount of the total variance in all models (conditional *R* ^2^ range = 0.62–0.78), suggesting that grouping by student and course section explained a considerable portion of the outcome variance. Demographic variables (Pell, first‐generation, honors college, GPA, and gender) were included as fixed effects but were not significantly related to belonging, research confidence, or research identity in our models. Though non‐significant by a small margin, first‐generation students reported smaller pre/post gains in belonging to major than their peers (*B* = −0.45, *p* = 0.067), which warrants further investigation.

**TABLE 2:** Linear mixed models to assess pre/post changes in research confidence and identity.

Predictors	Research confidence			Research identity		
	Estimates	CI	*p*	Estimates	CI	*p*
(Intercept)	5.48	5.23–5.72	**< 0.001**	4.99	4.41–5.58	**< 0.001**
Time (Post‐score)	0.57	0.41–0.74	**< 0.001**	0.75	0.55–0.95	**< 0.001**
Gender (Women & gender diverse)	−0.13	−0.39–0.13	0.330	−0.36	−0.82–0.09	0.116
Pell eligible (low income)	0.00	−0.38–0.38	0.998	0.29	−0.38–0.95	0.393
First‐generation	−0.08	−0.38–0.23	0.623	−0.29	−0.82–0.25	0.291
Cumulative GPA end of term	−0.12	−1.01–0.77	0.797	−0.47	−2.02–1.08	0.552
Honors	0.08	−0.27–0.44	0.648	0.03	−0.59–0.64	0.934
**Random effects**						
*σ* ^2^	0.36			0.55		
*τ*_00_	0.09_Student ID_			0.57_Student ID_		
	0.41_Class Section_			0.87_Class Section_		
ICC	0.58			0.72		
*N*	104_Student ID_			104_Student ID_		
	7_Class Section_			7_Class Section_		
Observations	208			208		
Marginal *R* ^2^/Conditional *R* ^2^	0.091/0.620			0.094/0.750		

*Note:* Values that are statistically significant (*p* < 0.05) are presented in bold.

**TABLE 3:** Linear mixed model to assess pre/post changes in belonging to course and major.

Predictors	Belonging—Course			Belonging—Major		
	Estimates	CI	*p*	Estimates	CI	*p*
(Intercept)	5.22	4.81–5.63	**< 0.001**	5.72	5.35–6.08	**< 0.001**
Time (Post‐score)	0.62	0.40–0.83	**< 0.001**	0.37	0.22–0.52	**< 0.001**
Gender (Women & gender diverse)	0.27	−0.19 – 0.72	0.251	0.28	−0.13 – 0.69	0.185
Pell eligible	0.10	−0.53–0.73	0.752	0.38	−0.19–0.94	0.185
First‐generation	−0.28	−0.82–0.26	0.307	−0.45	−0.94–0.03	0.067
Cumulative GPA end of term	−0.00	−0.61–0.60	0.989	0.01	−0.53–0.56	0.962
Honors	−0.98	−2.50–0.54	0.203	−0.41	−1.78–0.96	0.550
**Random effects**						
*σ* ^2^	0.45			0.22		
*τ*_00_	0.00_Student ID_			0.00_Student ID_		
	0.70_Class Section_			0.68_Class Section_		
ICC	0.61			0.76		
*N*	77_Student ID_			75_Student ID_		
	5_Class Section_			5_Class Section_		
Observations	154			150		
Marginal *R* ^2^/Conditional *R* ^2^	0.100/0.649			0.086/0.779		

*Note:* Values that are statistically significant (*p* < 0.05) are presented in bold.

### 4.4 Research Q2: How Do Research Confidence and Identity Differ Across Student Demographics Including Income, First‐Generation Status, Gender, and Prior Academic Achievement?

**FIGURE 3:**
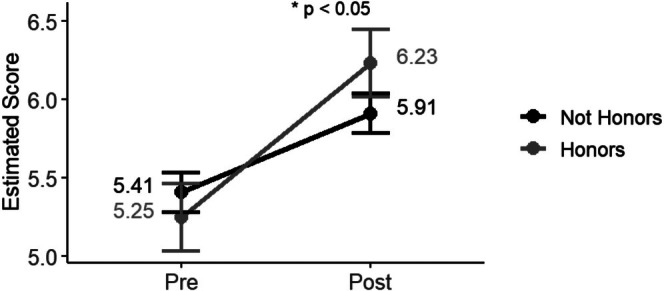
Research confidence estimated marginal means of pre‐post scores for honors and time interaction.

No demographic variables were significant in our global interaction model (Appendix S2: Table B1). In our models where we ran one interaction at a time, we did not find differences based on gender, first‐generation status, Pell eligibility, or GPA (Appendix S3: Tables C1, C3, C4, C5). However, honors status was significantly related to research confidence (Appendix S3: Table C2). While both honors and non‐honors students experienced pre/post gains, honors students reported steeper gains (Figure 3).

## 5 Discussion

This study demonstrates that a short, weekend‐long field course boosts students' sense of belonging, research identity, and research confidence. These findings contribute to the existing literature focused on the benefits of residential field experiences (Fedesco et al. 2020; Shaulskiy et al. 2022) and fill a key gap by adding to the limited literature on outcomes of short‐duration field courses. These findings suggest that a residential field course does not have to span multiple weeks, as is typical of the discipline, to foster research identity, confidence, and belonging. Short field courses can serve as an alternative or low‐stakes first step to field work for students who face barriers related to lengthier field courses, which is encouraging in terms of increasing equity in ecology (Morales et al. 2020; Ruskin et al. 2024).

Notably, despite EES 217 being only 48 h, we observed student outcomes that align with those resulting from multi‐week field courses (Easton and Gilburn 2012; Nicotra et al. 2022; Shaulskiy et al. 2022), which typically average seven weeks in duration (O'Brien et al. 2020). Shaulskiy et al. (2022) examined field courses ranging from 10 days to 7 weeks, with 78% being > 3 weeks, and found that students demonstrated significant gains in self‐reported research skills compared with those in on‐campus courses. The total effect of the field station setting on self‐reported research design and process skills reported by Shaulskiy et al. (2022) using serial mediation models was positive, similar in direction to our findings on research confidence. A study on student outcomes related to a 2‐week intensive residential field course reported that 76% of students reflected that the experience had fostered their science identity (Nicotra et al. 2022), compared with 65% of students gaining in research identity from pre/post in this study. However, our ability to compare directly to existing literature was challenged by the many different survey instruments and methods used.

Additionally, we found that short field experiences do not need to be situated within a semester‐long course to be effective. Incorporating overnight field work experiences into semester courses has been shown to foster science identity, deeper peer connections (Race et al. 2021), motivation, and learning gains (Fedesco et al. 2020). Our findings expand on this by illustrating that a standalone overnight field experience is related to positive pre/post gains in research identity, meaning field experiences can likely be impactful even in a short time frame. We recommend that future research on short, residential field courses assess content‐related outcomes, grades, and learning gains in addition to affective outcomes (e.g., Shinbrot et al. 2022).

Across the five demographic variables tested for interactions, we generally found that outcomes were not correlated with demographic variables. One exception was the pattern that honors students reported greater gains in research confidence than non‐honors students. We caution against over‐interpreting this result due to the small sample of honors students (*n* = 15). It is also possible that these differences stem from the distinct profile of an honors student, as they typically have a stronger high school academic preparation and different motivations to learn than non‐honors students (Cognard‐Black and Spisak 2019). Though we caution against generalizing this finding to other field courses, the instructors plan to consider this finding in future iterations of the course by exploring ways to further support student engagement and learning across all student groups.

Previous work has shown that field courses, as compared to lectures, increase self‐efficacy among all demographic groups, closing achievement gaps between underrepresented students and their peers (Beltran et al. 2020), which is promising for increasing ecology equity through field courses. We observed positive gains across most demographic variables, suggesting that the course supported students equitably. We recognize that our small sample size may have limited these analyses. It is possible that a greater sample size would provide the statistical power to observe differential effects based on demographic variables such as first‐generation status and gender. The statistical analyses showed a negative trend in belonging (major) for first‐generation (*b* = −0.45, *p* = 0.067) and though this was non‐significant, it warrants further research. First‐generation students face unique challenges in both university life (Pascarella et al. 2004) and in field courses (Fleischner et al. 2017), making it essential to study their experiences in more depth. We recommend utilizing purposeful sampling methods to better understand the unique barriers they face.

The short field course model can also be adapted to provide ecology field experiences to students in non‐ecology or non‐STEM majors. Increasing ecological literacy among non‐majors has been identified as a critical need in society (Byrne et al. 2025; Middendorf et al. 2020; Rodgers et al. 2024). A short, pass/fail field course such as EES 217 has the potential to attract students from a wide range of disciplines, particularly if designed around human‐environmental interactions, as suggested by Rodgers et al. (2024). Interdisciplinary field courses focusing on topics such as climate change or conservation policy could increase engagement for non‐majors. For example, researchers at the University of California Merced Natural Reserve System have created a short field course open to all majors aimed at reducing barriers to field work and have found that students, including non‐STEM majors, reported an increased sense of belonging to their institution and to field science (Brown et al. 2024). This is critical to engaging a wider range of students in ecological and conservation issues. Overall, the short field course model can be a promising addition to the undergraduate ecology curricula for both majors and non‐majors.

### 5.1 Limitations

This study has several limitations, one being the lack of a comparison or control group. Future research would benefit from comparing the results of short field courses to (1) longer field courses and (2) courses engaging in field activities on campus with similar goals, content, and instruments. This would help us to better understand the role of course duration in terms of student outcomes. Additionally, engaging students in similar course activities on campus would help us better understand the role of the residential aspect, or the location of the field course. For example, students could conduct a research project from start to finish over a 3‐day period on campus over spring break, and these results could be compared to students who participated in the residential field course. Future studies should also carefully select survey tools based on the existing literature to improve the direct comparability of future studies. Another limitation of this study was the potential impact of different instructors throughout the study period. It is possible that instructor characteristics and teaching practices had an effect on students; however, we aimed to reduce that effect by controlling for class section in the models.

Long‐term impacts were not assessed here as we only focused on the immediate effects at the conclusion of the course. As others have discussed, longitudinal studies on field course outcomes are lacking (e.g., Ward et al. 2021). However, these data would allow for us to better understand whether the positive impacts related to belonging, research identity, and confidence persist through time (e.g., 3 months later). Longitudinal data might also be helpful in understanding the relationship between field course participation and the likelihood of pursuing future research or graduate studies. For example, a study focused on graduate students found that field course participants had increased publication rates within 10 years of graduating (Arcila Hernández et al. 2023). We recommend that future work includes the measurement of long‐term impacts either by additional surveys or follow up interviews with students.

## 6 Conclusion

Our results extend prior work on the benefits of undergraduate field experiences by showing that a short, weekend‐long residential course delivers positive outcomes. This is promising for increasing equity in ecology as short field courses have the potential to reduce constraints such as costs, work and family obligations, and other barriers (e.g., Ruskin et al. 2024; Zavaleta et al. 2020). Additionally, short field courses may also be more accessible for instructors due to less time required and lower costs (Shinbrot et al. 2022). We do not suggest replacing traditional field courses altogether, but rather offering short field courses as one of the many field experience options for students. By broadening the range of accessible field courses we can ensure more students have the opportunity to engage in field work, as well as foster more inclusive pathways into careers in ecology.

## Author Contributions

**Holly C. White:** conceptualization (equal), data curation (equal), formal analysis (lead), investigation (lead), methodology (lead), visualization (lead), writing – original draft (lead). **Katharine J. Ruskin:** conceptualization (equal), formal analysis (supporting), funding acquisition (lead), investigation (supporting), methodology (supporting), project administration (equal), resources (equal), supervision (equal), writing – review and editing (equal). **Debra M. Allen:** conceptualization (equal), data curation (equal), funding acquisition (equal), project administration (equal), resources (equal), supervision (equal), writing – review and editing (equal). **Kayla B. McLagan:** conceptualization (equal), writing – review and editing (equal). **Sarah J. Nelson:** conceptualization (equal), writing – review and editing (equal). **Alison Jolley:** conceptualization (equal), investigation (supporting), methodology (supporting), supervision (equal), writing – review and editing (equal).

## Conflicts of Interest

The authors declare no conflicts of interest.

## Supporting information


**Appendix S1–S3:** ece372517‐sup‐0001‐AppendixS1‐S3.docx.

## Data Availability

All the required data is uploaded as Supporting Information. To ensure FERPA (Family Educational Rights and Privacy Act) compliance, the authors will share aggregated, deidentified data upon request.
