# Supplementary material for: Effects of a Weekend‐Long Field Course on Undergraduates' Confidence, Identity, and Belonging
Source: Ecol Evol. 2025 Dec 4;15(12):e72517. doi: 10.1002/ece3.72517 (PMC12676252; doi:10.1002/ece3.72517)
Supplement: Supplementary file 1 — Appendix S1–S3: ece372517‐sup‐0001‐AppendixS1‐S3.docx. [file ECE3-15-e72517-s001.docx]

**Appendix S1**

| Source | Survey items used in the study |
| --- | --- |
| Sense of belonging (adapted from Bollen & Hoyle, 1990) | Please indicate the extent to which you agree or disagree with the following statements.  [7-point Likert scale: Strongly disagree, disagree, somewhat disagree, neither agree nor disagree, somewhat agree, agree, strongly agree]  ***Belonging at major/discipline level***  [provide option for: “I do not have a major/discipline”]  1. I feel a sense of belonging to my major/academic discipline.  2. I feel that I am a member of my major/academic discipline.  3. I see myself as part of my major/academic discipline.  ***Belonging at course level***  1. I feel a sense of belonging to this course community.  2. I feel that I am a member of my course community.  3. I see myself as part of my course community. |
| Research self-efficacy  (adapted from Robnett et al., 2015) | Please indicate the extent to which you agree or disagree with the following statements.  [7-point Likert scale: Strongly disagree, disagree, somewhat disagree, neither agree nor disagree, somewhat agree, agree, strongly agree]  Research refers to any activity involving creativity production, exploration, or other research processes.  ***Research confidence***   1. I know how to relate the results from research to the work of others. 2. I know how to generate a research question to answer. 3. I know how to use research conducted by others to guide my research. 4. I am good at creating explanations for the results of a project. 5. I know how to develop theories (integrate the results of multiple research studies). 6. I am good at using research language and terminology. 7. I am good at using technical research skills.   ***Research identity***   1. I feel like I belong in research. 2. I am a researcher. |
|  |  |
| Demographics | Reminder: all responses are confidential and will NOT be shared with your instructor or peers.  What are your goals beyond your undergraduate degree? Please choose all that apply:  ● My goal is to go to graduate school for an advanced degree in a science-related field (including biology, chemistry, physics, mathematics, computer science, and psychology).  ● My goal is to go to graduate school for an advanced degree in a social science (including sociology, anthropology, economics, and political science).  ● My goal is to go to graduate school for an advanced degree in humanities or fine arts.  ● My goal is to earn a certification or degree that will qualify me for teaching.  ● My goal is to go to school for a medical degree (M.D. or other).  ● My goal is to go to a type of graduate education not mentioned above, such as law school.  ● My goal does not include graduate education for at least the near future.  ● Not applicable/Prefer not to answer.  With which gender do you identify?  ● Man  ● Woman  ● Gender non-binary  ● I prefer to self-identify: [open response]  ● I prefer not to answer  What is your sexual orientation/identity currently (check all that apply to you)?  ● Asexual  ● Bisexual  ● Gay  ● Lesbian  ● Pansexual  ● Queer  ● Straight/heterosexual  ● I prefer to self-identify: [open response]  ● I prefer not to answer  Think of this slider as representing where people stand in the United States. At 10 are people who are the best off - those with most money, education, and most respected jobs. At 1 are the people who are the worst off- those with the least money, education, and least respected of jobs or no job.  ● Where would you place your family as you were growing up on this scale? [slider that goes from 1 worst off to 10 best off]  Do you have at least one parent or guardian who has completed a graduate degree? (Masters, Doctoral or Professional degree, for example, MSW, JD, PhD)  ● Yes  ● No  ● I prefer not to answer  Is there any other relevant aspect of your personal identity that you would like to share? [open response] |

**Appendix S2**

Table B1. Global interaction model including all demographic predictors

|  | **Research Confidence** | | **Research Identity** | | **Belonging - Course** | | **Belonging - Major** | |
| --- | --- | --- | --- | --- | --- | --- | --- | --- |
| *Predictors* | *Estimates* | *CI* | *Estimates* | *CI* | *Estimates* | *CI* | *Estimates* | *CI* |
| (Intercept) | 5.55 ^***^ | 5.28 – 5.83 | 5.09 ^***^ | 4.48 – 5.70 | 5.18 ^***^ | 4.72 – 5.63 | 5.73 ^***^ | 5.34 – 6.12 |
| Time (Post-score) | 0.42 ^**^ | 0.11 – 0.73 | 0.56 ^**^ | 0.18 – 0.93 | 0.71 ^**^ | 0.25 – 1.17 | 0.35 ^*^ | 0.03 – 0.67 |
| Gender (Women & Gender Diverse) | -0.18 | -0.50 – 0.14 | -0.48 | -0.99 – 0.03 | 0.31 | -0.21 – 0.84 | 0.24 | -0.21 – 0.69 |
| Pell eligible (low income) | -0.04 | -0.51 – 0.43 | 0.05 | -0.69 – 0.80 | 0.03 | -0.70 – 0.76 | 0.39 | -0.23 – 1.00 |
| First-generation | -0.13 | -0.50 – 0.25 | -0.36 | -0.96 – 0.24 | -0.16 | -0.79 – 0.46 | -0.35 | -0.88 – 0.19 |
| Honors | -0.13 | -0.57 – 0.30 | 0.16 | -0.54 – 0.85 | 0.01 | -0.68 – 0.71 | 0.01 | -0.59 – 0.60 |
| Cumulative GPA End of Term | -0.19 | -1.28 – 0.91 | -1.00 | -2.73 – 0.74 | -0.90 | -2.65 – 0.86 | -0.25 | -1.75 – 1.25 |
| Time (Post-score)*  Gender (Women & Gender Diverse) | 0.10 | -0.27 – 0.47 | 0.23 | -0.22 – 0.69 | -0.10 | -0.62 – 0.43 | 0.07 | -0.29 – 0.44 |
| Time (Post-score)* Pell eligible | 0.09 | -0.46 – 0.63 | 0.47 | -0.20 – 1.14 | 0.13 | -0.60 – 0.86 | -0.01 | -0.52 – 0.49 |
| Time (Post-score)* First-generation | 0.10 | -0.34 – 0.54 | 0.15 | -0.39 – 0.69 | -0.23 | -0.85 – 0.39 | -0.21 | -0.64 – 0.22 |
| Time (Post-score)* Honors | 0.43 | -0.08 – 0.94 | -0.26 | -0.89 – 0.36 | -0.04 | -0.74 – 0.66 | 0.01 | -0.47 – 0.50 |
| Time (Post-score)* Cumulative GPA End of Term | 0.14 | -1.13 – 1.42 | 1.06 | -0.50 – 2.63 | -0.16 | -1.91 – 1.58 | -0.32 | -1.55 – 0.90 |
| **Random Effects** | | | | | | | | |
| σ^2^ | 0.36 | | 0.54 | | 0.47 | | 0.23 | |
| τ_00_ | 0.09 _Student ID_ | | 0.57 _Student ID_ | | 0.00 _Student ID_ | | 0.00 _Student ID_ | |
|  | 0.41 _Class Section_ | | 0.87 _Class Section_ | | 0.69 _Class Section_ | | 0.67 _Class Section_ | |
| ICC | 0.58 | | 0.72 | | 0.59 | | 0.75 | |
| N | 104 _Student ID_ | | 104 _Student ID_ | | 77 _Student ID_ | | 75 _Student ID_ | |
|  | 7 _Class Section_ | | 7 _Class Section_ | | 5 _Class Section_ | | 5 _Class Section_ | |
| Observations | 208 | | 208 | | 154 | | 150 | |
| Marginal R^2^ / Conditional R^2^ | 0.099 / 0.623 | | 0.099 / 0.752 | | 0.101 / 0.635 | | 0.087 / 0.770 | |
| *Note.* * p<0.05 ** p<0.01 *** p<0.001. Models include all main effects and interaction terms between post-score and gender, Pell eligibility, first-generation status, honors status, and cumulative GPA for student outcomes: research confidence, research identity, and course/major belonging. | | | | | | | | |

**Appendix S3**

Table C1. Interaction between gender and time on student outcomes

|  | **Research Confidence** | | **Research Identity** | | **Belonging - Course** | | **Belonging - Major** | |
| --- | --- | --- | --- | --- | --- | --- | --- | --- |
| *Predictors* | *Estimates* | *CI* | *Estimates* | *CI* | *Estimates* | *CI* | *Estimates* | *CI* |
| (Intercept) | 5.54 ^***^ | 5.27 – 5.80 | 5.08 ^***^ | 4.47 – 5.68 | 5.18 ^***^ | 4.73 – 5.62 | 5.74 ^***^ | 5.35 – 6.12 |
| Time (Post-score) | 0.45 ^**^ | 0.16 – 0.73 | 0.59 ^**^ | 0.24 – 0.94 | 0.71 ^***^ | 0.32 – 1.10 | 0.33 ^*^ | 0.06 – 0.61 |
| Gender (Women & Gender Diverse) | -0.22 | -0.53 – 0.09 | -0.49 | -0.99 – 0.02 | 0.33 | -0.18 – 0.84 | 0.25 | -0.19 – 0.69 |
| Pell eligible (low income) | 0.00 | -0.38 – 0.38 | 0.29 | -0.38 – 0.95 | 0.10 | -0.53 – 0.73 | 0.38 | -0.19 – 0.94 |
| First-generation | -0.08 | -0.38 – 0.23 | -0.29 | -0.82 – 0.25 | -0.28 | -0.82 – 0.26 | -0.45 | -0.94 – 0.03 |
| Honors | 0.08 | -0.27 – 0.44 | 0.03 | -0.59 – 0.64 | -0.00 | -0.61 – 0.60 | 0.01 | -0.53 – 0.56 |
| Cumulative GPA End of Term | -0.12 | -1.01 – 0.77 | -0.47 | -2.02 – 1.08 | -0.98 | -2.50 – 0.54 | -0.41 | -1.78 – 0.96 |
| Time (Post-score) *  Gender (Women & Gender Diverse) | 0.18 | -0.17 – 0.53 | 0.25 | -0.18 – 0.68 | -0.13 | -0.60 – 0.34 | 0.05 | -0.28 – 0.38 |
| **Random Effects** | | | | | | | | |
| σ^2^ | 0.36 | | 0.54 | | 0.45 | | 0.22 | |
| τ_00_ | 0.09 _Student ID_ | | 0.57 _Student ID_ | | 0.00 _Student ID_ | | 0.00 _Student ID_ | |
|  | 0.41 _Class Section_ | | 0.87 _Class Section_ | | 0.70 _Class Section_ | | 0.68 _Class Section_ | |
| ICC | 0.58 | | 0.73 | | 0.61 | | 0.76 | |
| N | 104 _Student ID_ | | 104 _Student ID_ | | 77 _Student ID_ | | 75 _Student ID_ | |
|  | 7 _Class Section_ | | 7 _Class Section_ | | 5 _Class Section_ | | 5 _Class Section_ | |
| Observations | 208 | | 208 | | 154 | | 150 | |
| Marginal R^2^ / Conditional R^2^ | 0.093 / 0.621 | | 0.095 / 0.751 | | 0.101 / 0.647 | | 0.086 / 0.777 | |
| *Note. * p<0.05 ** p<0.01 *** p<0.001* | | | | | | | | |

Table C2. Interaction between honors and time on student outcomes

|  | **Research Confidence** | | **Research Identity** | | **Belonging - Course** | | **Belonging - Major** | |
| --- | --- | --- | --- | --- | --- | --- | --- | --- |
| *Predictors* | *Estimates* | *CI* | *Estimates* | *CI* | *Estimates* | *CI* | *Estimates* | *CI* |
| (Intercept) | 5.51 ^***^ | 5.27 – 5.75 | 4.99 ^***^ | 4.41 – 5.58 | 5.22 ^***^ | 4.81 – 5.64 | 5.72 ^***^ | 5.36 – 6.08 |
| Time (Post-score) | 0.50 ^***^ | 0.33 – 0.68 | 0.75 ^***^ | 0.53 – 0.97 | 0.62 ^***^ | 0.39 – 0.85 | 0.36 ^***^ | 0.20 – 0.53 |
| Honors | -0.16 | -0.58 – 0.27 | 0.04 | -0.65 – 0.72 | 0.00 | -0.68 – 0.68 | -0.00 | -0.59 – 0.58 |
| Gender (Women & Gender Diverse) | -0.13 | -0.39 – 0.13 | -0.36 | -0.82 – 0.09 | 0.27 | -0.19 – 0.72 | 0.28 | -0.13 – 0.69 |
| Pell eligible (low income) | 0.00 | -0.38 – 0.38 | 0.29 | -0.38 – 0.95 | 0.10 | -0.53 – 0.73 | 0.38 | -0.19 – 0.94 |
| First-generation | -0.08 | -0.38 – 0.23 | -0.29 | -0.82 – 0.25 | -0.28 | -0.82 – 0.26 | -0.45 | -0.94 – 0.03 |
| Cumulative GPA End of Term | -0.12 | -1.01 – 0.77 | -0.47 | -2.02 – 1.08 | -0.98 | -2.50 – 0.54 | -0.41 | -1.78 – 0.96 |
| Time (Post-score) * Honors | 0.48 ^*^ | 0.02 – 0.94 | -0.02 | -0.60 – 0.56 | -0.02 | -0.63 – 0.60 | 0.03 | -0.40 – 0.46 |
| **Random Effects** | | | | | | | | |
| σ^2^ | 0.35 | | 0.55 | | 0.45 | | 0.22 | |
| τ_00_ | 0.09 _Student ID_ | | 0.57 _Student ID_ | | 0.00 _Student ID_ | | 0.00 _Student ID_ | |
|  | 0.42 _Class Section_ | | 0.87 _Class Section_ | | 0.70 _Class Section_ | | 0.68 _Class Section_ | |
| ICC | 0.59 | | 0.72 | | 0.61 | | 0.76 | |
| N | 104 _Student ID_ | | 104 _Student ID_ | | 77 _Student ID_ | | 75 _Student ID_ | |
|  | 7 _Class Section_ | | 7 _Class Section_ | | 5 _Class Section_ | | 5 _Class Section_ | |
| Observations | 208 | | 208 | | 154 | | 150 | |
| Marginal R^2^ / Conditional R^2^ | 0.099 / 0.633 | | 0.093 / 0.748 | | 0.100 / 0.646 | | 0.086 / 0.777 | |
| *Note. * p<0.05 ** p<0.01 *** p<0.001* | | | | | | | | |

Table C3. Interaction between first-generation status and time on student outcomes

|  | **Research Confidence** | | **Research Identity** | | **Belonging - Course** | | **Belonging - Major** | |
| --- | --- | --- | --- | --- | --- | --- | --- | --- |
| *Predictors* | *Estimates* | *CI* | *Estimates* | *CI* | *Estimates* | *CI* | *Estimates* | *CI* |
| (Intercept) | 5.48 ^***^ | 5.24 – 5.73 | 5.01 ^***^ | 4.42 – 5.59 | 5.21 ^***^ | 4.79 – 5.62 | 5.70 ^***^ | 5.34 – 6.07 |
| Time (Post-score) | 0.56 ^***^ | 0.37 – 0.74 | 0.72 ^***^ | 0.50 – 0.95 | 0.65 ^***^ | 0.42 – 0.89 | 0.40 ^***^ | 0.24 – 0.57 |
| First-generation | -0.12 | -0.50 – 0.26 | -0.37 | -0.97 – 0.23 | -0.17 | -0.79 – 0.44 | -0.35 | -0.87 – 0.18 |
| Gender (Women & Gender Diverse) | -0.13 | -0.39 – 0.13 | -0.36 | -0.82 – 0.09 | 0.27 | -0.19 – 0.72 | 0.28 | -0.13 – 0.69 |
| Pell eligible (low income) | 0.00 | -0.38 – 0.38 | 0.29 | -0.38 – 0.95 | 0.10 | -0.53 – 0.73 | 0.38 | -0.19 – 0.94 |
| Honors | 0.08 | -0.27 – 0.44 | 0.03 | -0.59 – 0.64 | -0.00 | -0.61 – 0.60 | 0.01 | -0.53 – 0.56 |
| Cumulative GPA End of Term | -0.12 | -1.01 – 0.77 | -0.47 | -2.02 – 1.08 | -0.98 | -2.50 – 0.54 | -0.41 | -1.78 – 0.96 |
| Time (Post-score) * First-generation | 0.09 | -0.35 – 0.53 | 0.17 | -0.37 – 0.71 | -0.21 | -0.80 – 0.39 | -0.21 | -0.62 – 0.20 |
| **Random Effects** | | | | | | | | |
| σ^2^ | 0.36 | | 0.55 | | 0.45 | | 0.22 | |
| τ_00_ | 0.09 _Student ID_ | | 0.57 _Student ID_ | | 0.00 _Student ID_ | | 0.00 _Student ID_ | |
|  | 0.41 _Class Section_ | | 0.87 _Class Section_ | | 0.70 _Class Section_ | | 0.68 _Class Section_ | |
| ICC | 0.58 | | 0.72 | | 0.61 | | 0.76 | |
| N | 104 _Student ID_ | | 104 _Student ID_ | | 77 _Student ID_ | | 75 _Student ID_ | |
|  | 7 _Class Section_ | | 7 _Class Section_ | | 5 _Class Section_ | | 5 _Class Section_ | |
| Observations | 208 | | 208 | | 154 | | 150 | |
| Marginal R^2^ / Conditional R^2^ | 0.092 / 0.618 | | 0.094 / 0.749 | | 0.101 / 0.648 | | 0.087 / 0.780 | |
| *Note. * p<0.05 ** p<0.01 *** p<0.001* | | | | | | | | |

Table C4. Interaction between Pell eligibility and time on student outcomes

|  | **Research Confidence** | | **Research Identity** | | **Belonging - Course** | | **Belonging - Major** | |
| --- | --- | --- | --- | --- | --- | --- | --- | --- |
| *Predictors* | *Estimates* | *CI* | *Estimates* | *CI* | *Estimates* | *CI* | *Estimates* | *CI* |
| (Intercept) | 5.48 ^***^ | 5.24 – 5.73 | 5.01 ^***^ | 4.43 – 5.60 | 5.23 ^***^ | 4.82 – 5.64 | 5.72 ^***^ | 5.35 – 6.08 |
| Time (Post-score) | 0.56 ^***^ | 0.38 – 0.73 | 0.71 ^***^ | 0.50 – 0.92 | 0.60 ^***^ | 0.37 – 0.83 | 0.37 ^***^ | 0.21 – 0.54 |
| Pell eligible (low income) | -0.07 | -0.53 – 0.40 | 0.10 | -0.65 – 0.84 | 0.03 | -0.68 – 0.75 | 0.40 | -0.21 – 1.01 |
| Gender (Women & Gender Diverse) | -0.13 | -0.39 – 0.13 | -0.36 | -0.82 – 0.09 | 0.27 | -0.19 – 0.72 | 0.28 | -0.13 – 0.69 |
| First-generation | -0.08 | -0.38 – 0.23 | -0.29 | -0.82 – 0.25 | -0.28 | -0.82 – 0.26 | -0.45 | -0.94 – 0.03 |
| Honors | 0.08 | -0.27 – 0.44 | 0.03 | -0.59 – 0.64 | -0.00 | -0.61 – 0.60 | 0.01 | -0.53 – 0.56 |
| Cumulative GPA End of Term | -0.12 | -1.01 – 0.77 | -0.47 | -2.02 – 1.08 | -0.98 | -2.50 – 0.54 | -0.41 | -1.78 – 0.96 |
| Time (Post-score) * Pell eligible | 0.13 | -0.41 – 0.67 | 0.38 | -0.28 – 1.04 | 0.14 | -0.53 – 0.81 | -0.04 | -0.51 – 0.43 |
| **Random Effects** | | | | | | | | |
| σ^2^ | 0.36 | | 0.54 | | 0.45 | | 0.22 | |
| τ_00_ | 0.09 _Student ID_ | | 0.57 _Student ID_ | | 0.00 _Student ID_ | | 0.00 _Student ID_ | |
|  | 0.41 _Class Section_ | | 0.87 _Class Section_ | | 0.70 _Class Section_ | | 0.68 _Class Section_ | |
| ICC | 0.58 | | 0.73 | | 0.61 | | 0.76 | |
| N | 104 _Student ID_ | | 104 _Student ID_ | | 77 _Student ID_ | | 75 _Student ID_ | |
|  | 7 _Class Section_ | | 7 _Class Section_ | | 5 _Class Section_ | | 5 _Class Section_ | |
| Observations | 208 | | 208 | | 154 | | 150 | |
| Marginal R^2^ / Conditional R^2^ | 0.092 / 0.618 | | 0.095 / 0.751 | | 0.100 / 0.646 | | 0.086 / 0.777 | |
| *Note. * p<0.05 ** p<0.01 *** p<0.001* | | | | | | | | |

Table C5. Interaction between GPA and time on student outcomes

|  | **Research Confidence** | | **Research Identity** | | **Belonging - Course** | | **Belonging - Major** | |
| --- | --- | --- | --- | --- | --- | --- | --- | --- |
| *Predictors* | *Estimates* | *CI* | *Estimates* | *CI* | *Estimates* | *CI* | *Estimates* | *CI* |
| (Intercept) | 5.48 ^***^ | 5.23 – 5.72 | 4.99 ^***^ | 4.41 – 5.58 | 5.22 ^***^ | 4.81 – 5.63 | 5.72 ^***^ | 5.35 – 6.08 |
| Time (Post-score) | 0.57 ^***^ | 0.40 – 0.74 | 0.75 ^***^ | 0.55 – 0.95 | 0.62 ^***^ | 0.40 – 0.83 | 0.37 ^***^ | 0.22 – 0.52 |
| Cumulative GPA End of Term | -0.38 | -1.45 – 0.69 | -0.97 | -2.68 – 0.74 | -0.85 | -2.55 – 0.86 | -0.32 | -1.79 – 1.16 |
| Gender (Women & Gender Diverse) | -0.13 | -0.39 – 0.13 | -0.36 | -0.82 – 0.09 | 0.27 | -0.19 – 0.72 | 0.28 | -0.13 – 0.69 |
| Pell eligible (low income) | 0.00 | -0.38 – 0.38 | 0.29 | -0.38 – 0.95 | 0.10 | -0.53 – 0.73 | 0.38 | -0.19 – 0.94 |
| First-generation | -0.08 | -0.38 – 0.23 | -0.29 | -0.82 – 0.25 | -0.28 | -0.82 – 0.26 | -0.45 | -0.94 – 0.03 |
| Honors | 0.08 | -0.27 – 0.44 | 0.03 | -0.59 – 0.64 | -0.00 | -0.61 – 0.60 | 0.01 | -0.53 – 0.56 |
| Time (Post-score) * Cumulative GPA End of Term | 0.53 | -0.66 – 1.72 | 1.01 | -0.45 – 2.46 | -0.26 | -1.82 – 1.30 | -0.19 | -1.28 – 0.91 |
| **Random Effects** | | | | | | | | |
| σ^2^ | 0.36 | | 0.54 | | 0.45 | | 0.22 | |
| τ_00_ | 0.09 _Student ID_ | | 0.57 _Student ID_ | | 0.00 _Student ID_ | | 0.00 _Student ID_ | |
|  | 0.41 _Class Section_ | | 0.87 _Class Section_ | | 0.70 _Class Section_ | | 0.68 _Class Section_ | |
| ICC | 0.58 | | 0.73 | | 0.61 | | 0.76 | |
| N | 104 _Student ID_ | | 104 _Student ID_ | | 77 _Student ID_ | | 75 _Student ID_ | |
|  | 7 _Class Section_ | | 7 _Class Section_ | | 5 _Class Section_ | | 5 _Class Section_ | |
| Observations | 208 | | 208 | | 154 | | 150 | |
| Marginal R^2^ / Conditional R^2^ | 0.093 / 0.620 | | 0.096 / 0.752 | | 0.100 / 0.646 | | 0.086 / 0.777 | |
| *Note. * p<0.05 ** p<0.01 *** p<0.001* | | | | | | | | |
